# Do "big guys" really die younger? An examination of height and lifespan in former professional basketball players

**DOI:** 10.1371/journal.pone.0185617

**Published:** 2017-10-02

**Authors:** Srdjan Lemez, Nick Wattie, Joseph Baker

**Affiliations:** 1 School of Kinesiology and Nutritional Science, California State University–Los Angeles, Los Angeles, United States of America; 2 Faculty of Health Sciences, University of Ontario Institute of Technology, Oshawa, Canada; 3 School of Kinesiology and Health Science, York University, Toronto, Canada; Leibniz Institute on aging—Fritz Lipmann Institute (FLI), GERMANY

## Abstract

While factors such as genetics may mediate the relationship between height and mortality, evidence suggests that larger body size may be an important risk indicator of reduced lifespan longevity in particular. This study critically examined this relationship in professional basketball players. We examined living and deceased players who have played in the National Basketball Association (debut between 1946–2010) and/or the American Basketball Association (1967–1976) using descriptive and Kaplan-Meier and Cox regression analyses. The cut-off date for death data collection was December 11, 2015. Overall, 3,901 living and deceased players were identified and had a mean height of 197.78 cm (± 9.29, Range: 160.02–231.14), and of those, 787 former players were identified as deceased with a mean height of 193.88 cm (± 8.83, Range: 167.6–228.6). Descriptive findings indicated that the tallest players (top 5%) died younger than the shortest players (bottom 5%) in all but one birth decade (1941–1950). Similarly, survival analyses showed a significant relationship between height and lifespan longevity when both dichotomizing [χ^2^ (1) = 13.04, *p* < .05] and trichotomizing [χ^2^ (2) = 18.05, *p* < .05] the predictor variable height per birth decade, where taller players had a significantly higher mortality risk compared to shorter players through median (HR: 1.30, 95% CI: 1.13–1.50, *p* < .05) and trichotomized tertile split (HR: 1.40, 95% CI: 1.18–1.68, *p* <. 05; tallest 33.3% compared to shortest 33.3%) analyses. The uniqueness of examining the height-longevity hypothesis in this relatively homogeneous sub-population should be considered when interpreting these results. Further understanding of the potential risks of early mortality can help generate discourse regarding potential at-risk cohorts of the athlete population.

## Introduction

A recent article by ESPN senior writer Jackie MacMullan entitled “*Larry Bird will die young*. *Just ask him”* highlights a growing concern and “fatalistic view” of lifespan longevity amongst former professional basketball players [1]. Certainly, the death of a former teammate or competitor can often initiate one’s own contemplation of mortality, and recent young deaths of former “big guy” National Basketball Association (NBA) players (e.g., Anthony Mason, Darryl Dawkins and Moses Malone) may accentuate this perception.

The premise that larger body size leads to reduction in lifespan longevity has generally been substantiated through scientific research over the past 40 years. For example, Samaras’ research suggests smaller body size is generally better for one’s health, and is supported by robust cross-cultural findings of average lifespan reduction with increasing height observed in groups such as deceased American male veterans [2], French males and females who died before the year 1861 [3, 4] and males born in Sardinia, Italy between 1866 and 1915 [5]. Thus, Samaras’ overarching conclusion suggests health practitioners should de-emphasize the association between wellbeing and increased height [6], despite this being somewhat counterintuitive to society’s belief of increased height being a by-product of a healthy lifestyle [7]. Nevertheless, the relationship between height and quality of life (particularly early to midlife) and eminence/higher social status may be less clear and different than the height-mortality link, where quality of life is more socially constructed and mortality is more biologically linked to health.

### Biological mechanisms

While the biological reason for the relationship between height and lifespan longevity in humans is not yet fully understood [8], it is difficult to ignore the potential profound effect of genetics on lifespan longevity. A study by He and colleagues [8] on 8,006 American men of Japanese ancestry found height was positively associated with mortality, and perhaps of more interest, they were the first to conclusively link the “longevity gene” *FOX03* to smaller body size and greater lifespan longevity in humans. In addition, Samaras [9] provided a summary of studies illuminating proposed biological mechanisms that underpin superior longevity in smaller humans. Various biological and physiological parameters change with increases in body size [9], and some of the most robust explanations for this apparent inherent detriment of greater height includes reduced cell replication potential in old age to maintain body tissues and organs [6] and higher incidence of DNA and free radical damage [10]. For example, a professional basketball player likely has trillions more cells compared to a gymnast, and as a consequence, cell replication required after cumulative exposure to stresses can ultimately lower the ability of healthy cells to replace the damaged ones later in life since regeneration is limited, leading to higher incidences of chronic diseases such as cancer and cardiovascular disease from greater exposure to carcinogens [11, 12]. In addition, organs such as the brain, liver and kidneys become disproportionate in size in taller individuals, which reduces their functional capacity relative to body mass (i.e., shorter individuals will have greater cell reserve over time) [6, 11].

Although a sizeable amount of evidence suggests that larger body size *independently* reduces longevity (e.g., [13]), it also important to recognize confounders of this relationship that affect biological parameters independent of body size characteristics, such as differences in genotypes, socioeconomic status (SES), education, medical care, relative weight, hygienic practices, nutrition, and lifestyle choices such as engaging in regular exercise and avoiding smoking [6, 8, 14]. Further, Samaras [6] has suggested that height generally explains less than 10% of the proportion of variance regarding longevity, and He and colleagues [8] surmise that the lack of consensus on how height affects lifespan longevity is likely due to the impact of these extraneous variables. For example, from a caloric restriction standpoint, Okinawan Japanese are relatively shorter and consume significantly fewer calories than mainland Japan, and the residents of the island have historically had the longest life expectancy, highest prevalence of centenarians per capita, and the lowest mortality from chronic diseases such as cardiovascular disease, cancer, and diabetes in the developed world [15, 16]. From a morbidity standpoint, a more recent study examining 144,701 postmenopausal women found a positive association of height with risk of *all* cancers [17]. In contrast, however, Gavrilov and Gavrilova [18] did not find an association between height and survival to age 100 years, reporting the average height of centenarians at ~50^th^ percentile, and other researchers have found height to be *inversely* associated with cardiovascular disease mortality risk (e.g., [19]). Thus, conflicting findings in epidemiological studies on height and health reflect the inherent complexity of controlling for various known and unknown biological processes [8] and the difficulty gaining access to official death certificates produced by government officials, such as coroner/medical examiner reports, which may limit analyses to “all-cause” rather than cause-specific deaths.

### Athletes

Compared to the general population there has been much less investigation into the relationship of height and lifespan longevity in athletes. Samaras [20] provided a summary of studies that have examined athletes, and a decrease in longevity per centimetre of height (years/cm) was reported in Finnish (–0.49) and Harvard (–0.70) athletes, and baseball (–0.35) and football (–0.81) players. While each of these athlete samples had a negative correlation between height and longevity, research by Lawler, Lawler, Gibson and Murray [21] examined mortality outcomes in professional basketball players, and Cox proportional hazards survival analyses did not reveal height to be a significant predictor of lifespan longevity in their crude regression model (Hazard Ratio (HR) 1.01, 95% Confidence Interval (CI): 0.96–1.05). Therefore, there appears to be conflicting evidence on the relationship between height and lifespan longevity in athletes from different sports.

The purpose of this study was to extend this research and provide a more in-depth critical analysis on the time-dependent variable of height on a population of athletes generally regarded as having superior height relative to the rest of the population, such as professional basketball players. Considering the recent media speculation regarding the concern of NBA “big guys” dying younger, a more up-to-date study to Lawler and colleagues’ [21] examination into the mortality outcomes of NBA and American Basketball Association (ABA) players was conducted, since they included players as of the 2004–2005 NBA season and calculated deaths as of December 31, 2011. This study exclusively measured height and its role on lifespan longevity, despite the expression of an individual’s longevity profile being affected by several variables. However, if height indeed influences longevity independently, deceased professional basketball players represent a promising group to further investigate this phenomenon in given their general exceptional height and relative homogeneity of other confounders such as affluence (particularly in the more recent decades), where the higher SES/social status may result in less confounding by factors such as ethnicity. We hypothesized that when adjusting for birth decade, exceptionally taller players will have died at relatively younger ages.

## Methods

### Population

The population of this study was comprised of living and deceased players who played in the NBA (debut between 1946–2010) and/or the ABA (1967–1976). A large proportion of players who debuted in the 2009–10 season were still active at the time of this study. The cut-off date for death data collection was December 11, 2015. Data were collected from publicly available sources, and this study had institutional ethics approval.

### Data collection

To compile our data, basketball-reference.com was the primary source used for retrieving players’ lifespan longevity and anthropometric information. Wikipedia.org was accessed as a supplemented source in the case of incomplete date of birth and/or death data (<10 cases). Twenty percent of the lifespan longevity and anthropometric data were cross-referenced in the official NBA encyclopedia [22], which confirmed complete agreement between data sources.

### Variables

Players were coded as living or deceased and height was converted into centimetres (cm). Height was dichotomized through a median split for each birth decade (for the entire living and deceased sample) to categorize players into “below median height” or “above median height.” In addition, height was also trichotomized into tertiles (i.e., split the predictor variable height into three parts) by calculating height ‘frequencies’ and ‘percentiles’ per birth decade to determine the distribution of height values (i.e., ‘cumulative percent’ of height) and percentile demarcations. Players were then categorized into ‘first third,’ (≤ 33.3%) ‘second third,’ (33.4% to 66.5%) and ‘last third’ (≥ 66.6%) height groups accordingly, where the ‘first third’ group contained the shortest players and the ‘last third’ group contained the tallest players. This was done in order to provide a more specific measure of height on mortality outcomes.

### Statistical analyses

Data were analyzed using 1) descriptive and 2) Kaplan-Meier and Cox regression survival analyses. Since average height was liable to change over the time-course of the athletic population studied, it was analyzed by percentiles for separate birth decades of players. In addition, lifespan results were compared to males from the United States (US) general population using life expectancy data from the latest vital statistics report from the Centers for Disease Control (CDC) [23] and from Noymer and Garenne’s [24] study on mortality differences in the US. For the survival analyses, a t-test median and trichotomized tertile split of height per birth decade was performed to also account for the changing anthropometric birth cohort effects. Analyses were performed using SPSS version 24, and statistical significance was defined as *p* < 0.05, at the 95% CI.

## Results

### Descriptives

Overall, 3,901 living and deceased NBA/ABA players were identified and had a mean height of 197.78 cm (± 9.29, Range: 160.02–231.14), and of those, 787 were identified as deceased as of December 11, 2015. The mean height of the deceased NBA/ABA player population was 193.88 cm (± 8.83, Range: 167.64–228.60). Table 1 shows the mean ages of death per birth decade, displayed by percentile height (bottom 5%, bottom 10%, 50^th^ percentile, top 10% and top 5%). The 5^th^ percentile included the shortest players whereas the 95^th^ percentile included the tallest players. A linear increase in mean height was observed for both living and deceased players born in the earliest decades to the most recent decades, which supports the commonly held view that the evolution of basketball players (and other athletes) is predicated on them becoming bigger, faster and stronger.

**Table 1:** Mean (*M*) ages of death per birth decade, by percentile height.

	Living	Deceased	Height percentiles, calculated for each birth decade separately				
Birth Decade (*(n)*	*M* (height in cm)	*M* (height in cm)	5^th^	10^th^	50^th^	90^th^	95^th^
≤ 1920^1^ (109)	177.80 (*n* = 2; ± 3.59)	76.71 ± 12.41 (188.65)	80.66 ± 10.88	77.12 ± 12.25	76.82 ± 12.80	74.41 ± 12.02	75.96 ± 16.25
1921–1930 (359)	189.66 (*n* = 82; ± 7.81)	75.29 ± 11.92 (191.49)	76.57 ± 12.64	76.78 ± 11.69	75.71 ± 10.86	73.49 ± 11.37	72.46 ± 11.05
1931–1940 (111)	194.37 (*n* = 162; ± 6.88)	66.19 ± 13.78 (196.88)	66.01 ± 8.46	66.42 ± 15.30	66.48 ± 13.24	65.73 ± 13.34	62.00 ± 14.28
1941–1950 (118)	196.72 (*n* = 570; ± 8.29)	56.69 ± 12.47 (197.21)	54.81 ± 14.84	56.34 ± 13.57	57.49 ± 12.43	54.67 ± 14.56	56.79 ± 14.37
1951–1960 (53)	198.99 (*n* = 580; ± 8.38)	51.24 ± 9.60 (198.93)	56.71 ± 3.81	56.70 ± 5.19	48.92 ± 10.60	54.17 ± 5.51	52.36 ± 5.72
≥ 1961^1^ (37)	^2^	39.61 ± 9.16 (205.67)	41.43 ± 11.87	36.98 ± 9.19	39.17± 9.05	39.28 ± 9.21	39.70 ± 8.97
Total (787)		68.11 ± 16.03 (193.88)	75.11 ± 13.07	74.58 ± 13.21	72.32 ± 13.97	59.01 ± 16.50	56.66 ± 16.48

^1^The earliest birthdate was in 1902, and the most recent was in 1982

^2^1961–1970: 199.50 cm (*n* = 681; ± 9.31); 1971–1980: 200.56 cm (*n* = 597; ± 9.53); 1981–1990: 200.95 cm (*n* = 440; ± 9.19)

Since the inaugural season of the NBA was not until 1946, a high majority of deceased players in this population were subjected to dying prematurely; therefore, the influence of height on lifespan longevity was interpreted by lifespan differences *within* the same decade of birth, rather than between decades. Descriptively, the tallest players (top 5%) died younger than the shortest players (bottom 5%) in all but one birth decade (1941–1950). In total, there was a negative linear relationship between height and lifespan; the shortest players lived the longest (75.1 years) and the tallest players lived the shortest (56.6 years). Interestingly, it appears that the robustness of this effect diminished in players born in more recent decades. More specifically, for players born in 1940 or earlier, the mean ages of death for the tallest players were below the mean ages of death for all players born within the same respective decade (e.g., players in the 95^th^ percentile of height born between 1931 and 1940 had a mean age of death of 62 years compared to 66.1 years for *all* players born during the same time span); however, this effect reversed for those born in 1941 or later (i.e., the tallest players lived slightly longer on average than the mean age of death for each birth decade). The large overall discrepancy in lifespan longevity between shorter and taller players is likely a result of the larger sample sizes of deceased players born in 1940 or earlier (n = 579) that found shorter players to live longer compared to more recently (≥ 1941; n = 208).

Given that our study included deceased former players as of December 11, 2015, only a small proportion had the opportunity to live a full life (i.e., to ‘old age’). Therefore, we compared the mean ages of death for players born in 1920 or earlier and between 1921–1930 to the life expectancies at birth and in 2013 (most recent data) for males from the US general population. The life expectancies at birth in the male US general population were 46.3 years in 1900, 48.4 years in 1910, 53.6 years in 1920 and 58.1 years in 1930 [24]. As expected, considering the players all survived infancy and childhood (i.e., they survived until entering their career), the mean ages of death of the NBA/ABA player population far exceeded these estimates; players born in 1920 or earlier died at 76.7 years on average, and 75.2 years on average for those born between 1921 and 1930. Further, the tallest players (95^th^ percentile) had similar ages of death to the mean age of death for all players in their respective birth decades (e.g., players in the 95^th^ percentile of height born in 1920 or earlier had a mean age of death of 75.9 years compared to 76.7 years for *all* players born during the same time span). Interestingly, when compared to the 2013 referent life expectancy of 76.4 years [23], players’ mean ages of death were very similar, with the largest discrepancies found in players born in 1920 or earlier who were in the bottom 5^th^ percentile in height (lived 80.6 years on average) and those born between 1921 and 1930 that were in the top 5^th^ percentile in height (lived 72.4 years on average).

### Survival analyses

**Fig 1:**
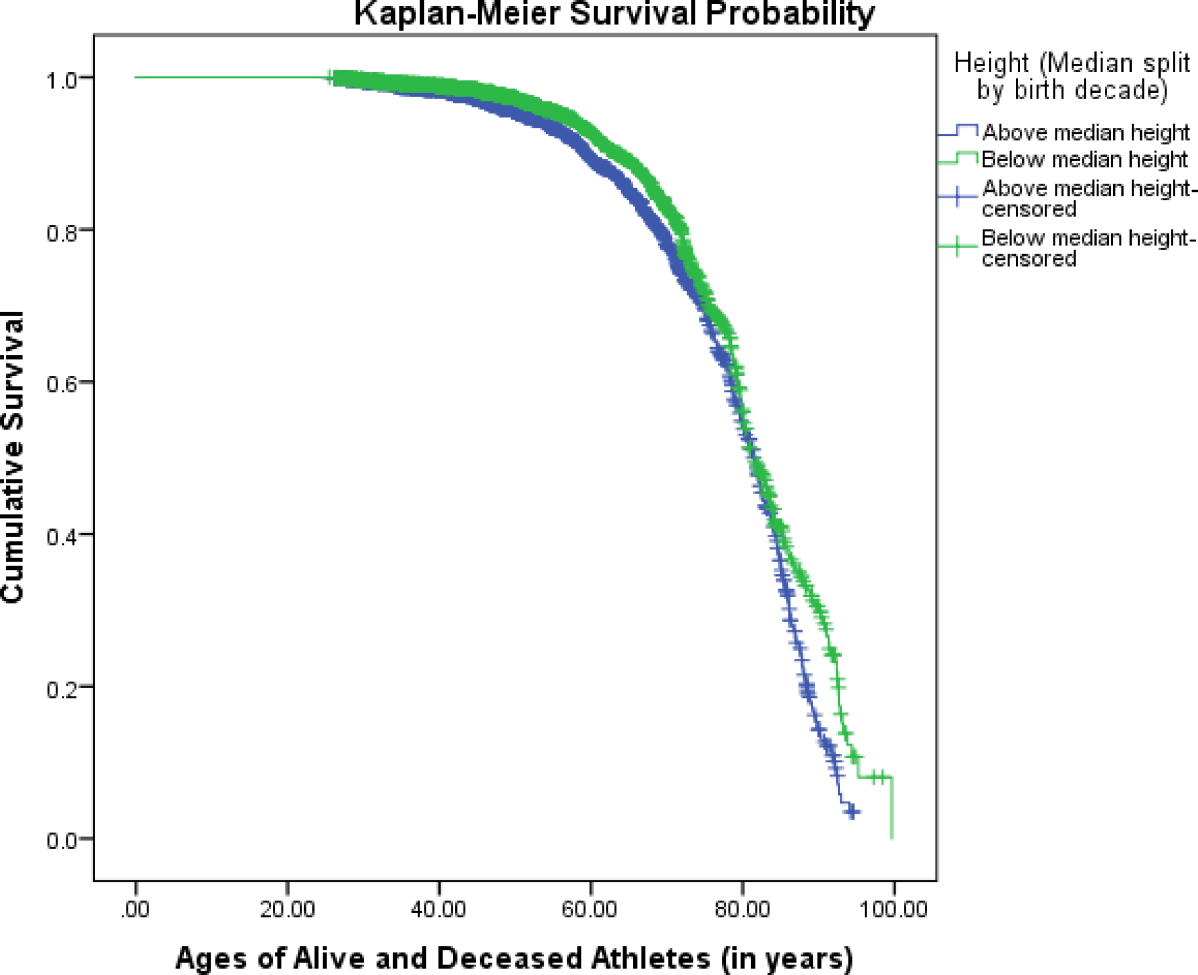
Kaplan-Meier survival probability, by above and below median height per birth decade [χ^2^ (1) = 13.04, *p* < .05].

Overall, data from 3,901 living and deceased players who debuted between 1946 and 2010 were included in this analysis. Fig 1 illustrates a survival curve grouped by above and below median height per birth decade. Kaplan-Meier survival analysis found a significant relationship between height and lifespan longevity in former NBA/ABA players [χ^2^ (1) = 13.04, *p* < .05]. In addition, Cox regression analysis found taller players to have a significantly higher mortality risk compared to shorter players (HR: 1.30, 95% CI: 1.13–1.50, *p* < .05).

**Fig 2:**
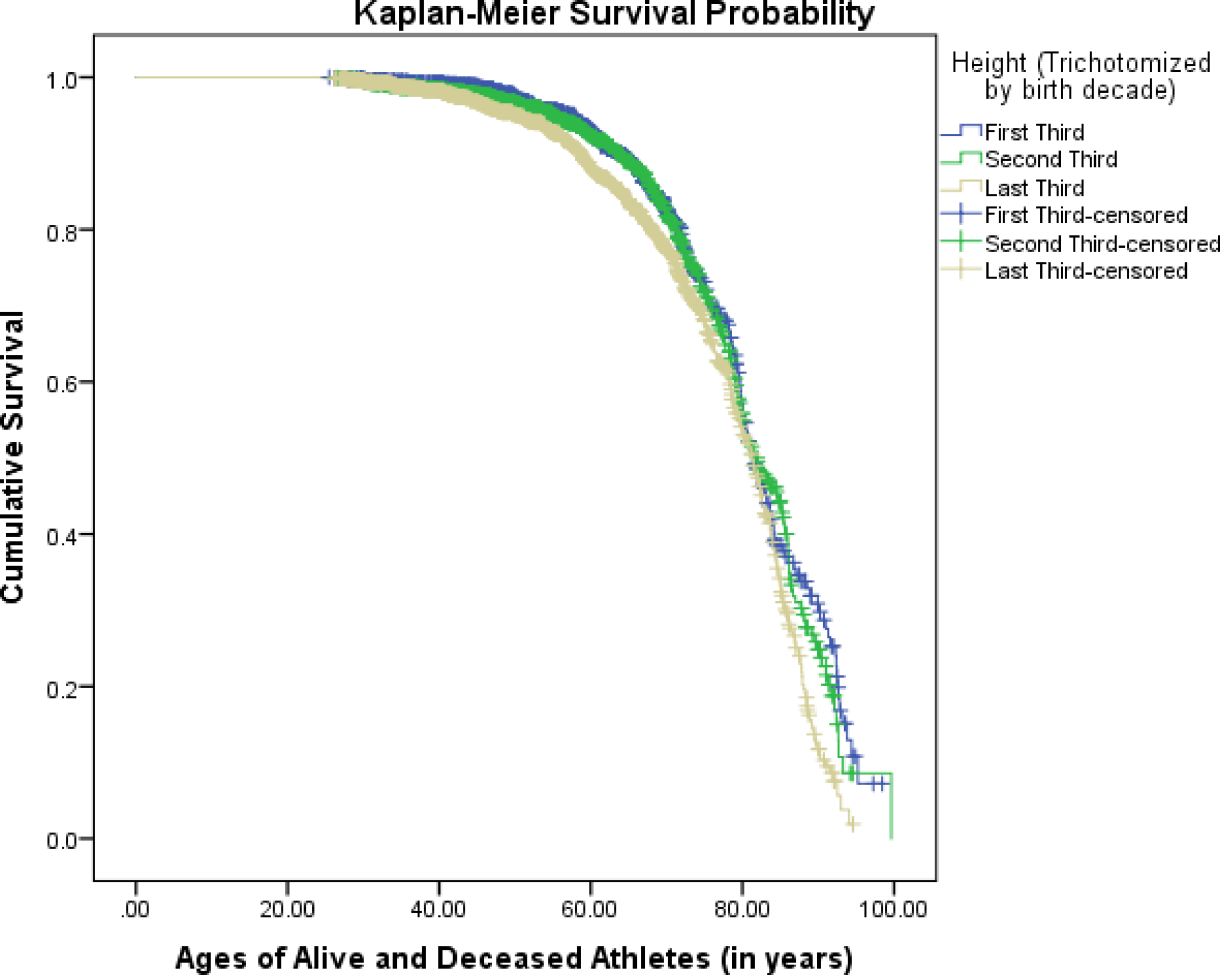
Kaplan-Meier survival probability, by ‘first third,’ ‘second third,’ and ‘last third’ height groups per birth decade [χ^2^ (2) = 18.05, *p* < .05].

Fig 2 illustrates a survival curve categorized by ‘first third,’ ‘second third,’ and ‘last third’ height groups per birth decade. Kaplan-Meier survival analysis also found a significant relationship between height and lifespan longevity after trichotomizing the predictor variable height into tertiles [χ^2^ (2) = 18.05, *p* < .05]. Likewise, Cox regression analysis found players who were in the ‘last third’ height group to have a significantly higher mortality risk compared to players who were in the ‘first third’ (i.e., shortest) height group (HR: 1.40, 95% CI: 1.18–1.68, *p* <. 05). There was no statistically significant difference in mortality risk between the ‘second third’ and ‘first third’ height groups (HR: 1.08, 95% CI: 0.89–1.30, *p* > .05). When referent groups were reversed (i.e., tallest player group used as referent), the mortality risk difference remained statistically significant when comparing the shortest player group to the tallest (HR: 0.76, CI 95%: 0.65–0.90); however, the mortality risk of the middle height group was also now statistically significant when compared to the tallest player group (HR: 0.71, 95% CI: 0.59–0.84).

## Discussion

The purpose of our study was to examine the influence of height on the lifespan longevities of professional basketball players, and we hypothesized that the relatively taller players would die at younger ages. Our hypothesis was supported through our key descriptive finding which indicated that there was an overall negative linear relationship between height and longevity where the tallest players died earliest on average. Interestingly, there was small lifespan *favourability* for the tallest players born in 1941 or later (in relation to the mean ages of death per birth decade); however, we postulate that this counterintuitive trend was likely a result from small sample sizes that may be skewing our findings. More specifically, 45.6% (n = 359) of the deceased players were born between 1921 and 1930, whereas only 6.7% (n = 53) were born between 1951 and 1960 and 4.7% (n = 37) after 1960. In addition to our descriptive analyses, Kaplan-Meier and Cox regression analyses also supported our hypothesis, where taller players had significantly higher mortality risk compared to shorter players, particularly those who belonged in the top 33.3% of height in their respective birth decade.

Our descriptive analyses allowed us to explore the nuances of lifespan longevity for the exceptionally tall cohorts (i.e., 95^th^ and 90^th^ percentiles). Therefore, in the present sample, the tallest players appear to die relatively younger than the shortest players, although findings for players born in the earlier decades may have more meaningful significance since they had the opportunity to live a full life. The lifespan differences in the later born players were likely confounded by more unnatural causes of death rather than the suggested negative biological dispositions of increased height. This hypothesis is supported by Lemez, Wattie, and Baker [25], who reported that the leading cause of death in *active* NBA players was car accidents, although only 10 cases of premature death were noted in this athlete group.

More importantly, a high majority of participants in professional basketball are taller than the average height of males in the general population, including players even in the bottom 10^th^ percentile of height. For example, since 1970, the average NBA player height has only dropped below 78 inches (198.12 cm) once (in 2010) [26]. Interestingly, it has also been suggested that artificial selection will continue in sports, where scouts will continue to look across the globe for taller players even though the industrial world appears to be past its growth spurt [27]. Although taller players appear to die younger relative to shorter players, we found the mean ages of death in the 95^th^ percentile in height of players born in 1930 or earlier to be similar to the life expectancies at birth in 2013 for males from the US general population (and still far superior to what their life expectancies estimated at the time of birth were, although time of birth estimates include those who die very young). Lawler and colleagues [21] suggested that the reasons for particular mortality outcomes for basketball players are likely to be multifactorial, such as the income, life experience, and SES incurred from being a professional athlete which allow for greater life quality such as better medical care. In addition, it is also important to consider the likelihood that height itself is associated with lifespan longevity, or whether it is simply an indicator of other underlying biological processes. These reasons, accompanied with exceptional height being such a rarity in the general population, complicate our understanding of health in individuals with heights in the highest percentiles. In fact, in David Epstein’s *The Sports Gene*, he states,

“An American man who is seven feet tall is such a rarity that the CDC does not even list a height percentile at that stature. But the NBA measurements combined with the curve formed by the CDC’s data suggest that of American men ages twenty to forty who stand seven feet tall, a startling 17 percent of them are in the NBA *right now*. Find six honest seven-footers, and one will be in the NBA.”

–([28], p. 131–132)

We calculated only 2% of the entire deceased player population to be seven feet (16/787; 213.36 cm), with a majority being born between 1961 and 1970 (9/16). Therefore, as professional athletes continue to become taller, more data will become available for future research to explore the rates and causes of death in the exceptionally tall athletes, which will provide us with more complete knowledge to delineate the reasons behind the potential risks involved with increased height, or whether being a professional athlete is enough to offset the apparent health detriments from larger body size.

### Limitations

First, a potential limitation of the height variable was that a median split of height by birth decade was used to measure mortality outcomes, which may have been too crude of a measurement of height and prevented investigation of potential non-linearity. However, while we used a more specific measure through a trichotomized tertile split (i.e., 33.3% × 33.3% × 33.3%) to circumvent this issue, the smaller sample sizes may have reduced statistical power, particularly in the deceased samples from more recent decades. This was further complicated by the players’ overall exceptional height; nearly all data were above the 90^th^ percentile for general US population height, making any-cut off point arbitrary. Second, since living players were included in the survival analysis, 79.8% of the NBA/ABA player population was censored, which may have limited some of the meaningfulness of the survival analyses results, although these findings were supported descriptively. Last, while the focus of this study was to critically examine the relationship between height and lifespan longevity, many other socially constructed or biological variables could have influenced mortality outcomes. Conversely, if height is considered a unique predictor of death, then controlling for variables such as decade of birth may not be as important.

## Conclusion

This study found that taller NBA/ABA players died earlier than shorter NBA/ABA players; however, the uniqueness of examining the height-longevity hypothesis in a relatively homogeneous population in terms of confounding factors should be considered when interpreting these results. As many players have superior height compared to the age- and sex-matched average height of the US general population, there appears to be a curvilinear relationship between height and longevity where the magnitude of mortality risk decreases past a certain threshold (i.e., for players in the 95^th^ percentile of height born in 1941 or later). However, smaller sample sizes in the younger players may have been driving this effect, and it is unknown whether this relationship is more monotonic in athletes. Further, Lemez and Baker’s [29] review suggested that elite athletes live longer, despite most of them likely being taller than individuals from the general population. Further evidence can inform and clarify our understanding of potential risks of early mortality of at-risk cohorts of the athlete population.

## Perspective

From a general population perspective, it is unclear whether there is a threshold for the apparent longevity benefits from having smaller body size (i.e., do the same benefits apply to persons who are in the 5^th^ percentiles of height and weight?). Continued research on the height-longevity hypothesis is needed in both athlete and non-athlete samples to demystify the nuances of this relationship, such as including cause of death data and examining the mortality outcomes of the smallest individuals.
